# Variable Clinical Phenotypes of α-Thalassemia Syndromes

**DOI:** 10.1100/tsw.2009.69

**Published:** 2009-07-13

**Authors:** Sylvia Titi Singer

**Affiliations:** Hematology/Oncology Department, Children's Hospital and Research Center (CHRCO), Oakland, CA, United States

**Keywords:** α-thalassemia, α-globin mutations, hemoglobin H, hemoglobin H-constant spring

## Abstract

Genetic mutations of the α genes are common worldwide. In Asia and particularly Southeast Asia, they can result in clinically significant types of α-thalassemia, namely hemoglobin (Hb) H disease and Hb Bart's hydrops fetalis. The latter is generally a fatal intrauterine condition, while Hb H disease results in clinical complications that are frequently overlooked. The high prevalence of the carrier state and the burden of these diseases (and other α-thalassemia variants) call for more attention for improved screening methods and better care.

## INTRODUCTION

α-Thalassemias are very common. The more severe forms are largely restricted to Southeast Asia (SEA) and China, and were thought to pose less of a worldwide problem than the β-thalassemias. However, increased migration patterns from these areas have resulted in α-thalassemia becoming a more global problem (Fig. 1). The carrier rate of the common α-globin gene deletion (--/SEA) is reported to be 3–14% in various areas in Asia, including 4.6% in Northern Thailand, 4.1% in Hong Kong, and 4.1% in Guangdong Province in China[1,2]. The frequency of the nondeletional α gene mutation, Constant Spring, was found to be between 1 and 6% in various areas of Southeast Asia, with the highest rate in Northeastern Thailand and Laos[3]. In North America and Europe, this emigration pattern resulted in the manifestation of clinical phenotypes, many involving α-thalassemia mutations that were previously less often recognized. The North American cross-sectional study identified 132 patients out of a total of 721 analyzed (18%) with clinically significant α-thalassemia syndromes[4]. This high incidence should spur more efforts to develop programs for newborn and prenatal screening, and for understanding genotype-phenotype correlations[4,5]. In Asia, comprehensive control programs were also developed, aimed to limit the numbers of new affected births and to prolong life in affected individuals; however, such programs have been successful in a minority of countries and have had little global impact. More widespread prevention methods, and management of affected children and adults with moderately severe forms of α-thalassemia, are needed in regions where the disease is prevalent[1].

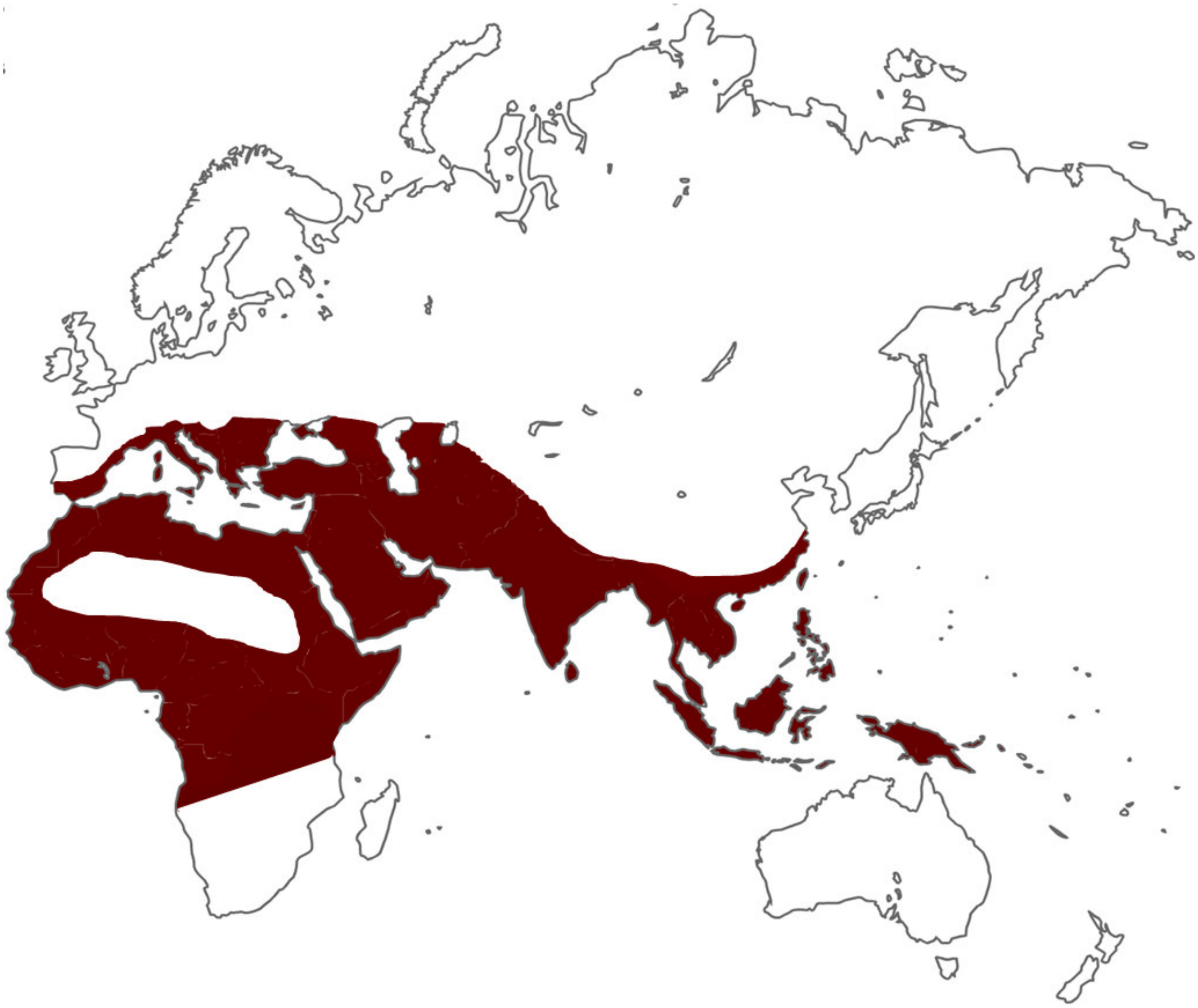

**FIGURE 1.** World distribution of α-thalassemia. Affected locations are represented by dark red areas (■).

In the clinical setting, improved screening methods, along with better management, have underscored the different patterns of α-thalassemia and diagnosed complications that were previously less well recognized[6]. As technology improved, successful attempts to treat fetuses and infants affected with α-thalassemia major, generally considered fatal, have been reported.

It is important to recognize the main types of α-thalassemia and the potential for genotypic combinations leading to different clinical symptomatology. Such knowledge will increase awareness, and improve monitoring and management of the various subtypes of α-thalassemia.

## UNDERSTANDING α-THALASSEMIA: PATHOPHYSIOLOGY AND CLASSIFICATION

β-Globin–chain synthesis is fully activated after birth, causing β-thalassemia syndromes to be expressed after birth, usually after γ-globin chain synthesis declines during the first months of life. This results in excess α-chains, which have a deleterious effect on the red cell and on erythropoiesis[7]. In contrast, α-

chains are part of the fetal and adult hemoglobin, and therefore can manifest in both fetal and postnatal life[8]. Defective α-globin production results in excess γ- and β-globin chains, each able to form soluble tetramers: γ4 (Hb Bart's) and β4 (Hb H). These tetramers have very high oxygen affinity and are therefore useless for effective oxygen delivery.

The α-globin gene is duplicated on each copy of chromosome 16, so there are a total of four α-globin genes (two genes per haploid; α1 and α2) in a normal genotype (αα/αα) (Fig. 2). They appear to have a higher tendency for deletions than most of the mutations affecting the β-globin gene, which frequently result from one or more nucleotide substitution or deletions. The production of α-globin decreases in proportion to the number of genes deleted[9,10]. The clinical severity of α-thalassemia relates to the number of genes affected out of the four α genes and correlates with the presence of the γ4 or β4 tetramers, and the extent to which they reduce erythropoiesis and red cell survival[11]. Recently, a correlation between α/β-globin mRNA ratio and disease severity was shown[12](Table 1). The most serious and frequently fatal of the α-thalassemia syndromes is that of the four gene–deletion syndrome, hydrops fetalis[13]. Deletion of three α genes, resulting in hemoglobin H (Hb H) disease, has a mild to moderately severe phenotype, while deletion of two or one α-globin genes has no clinical significance.

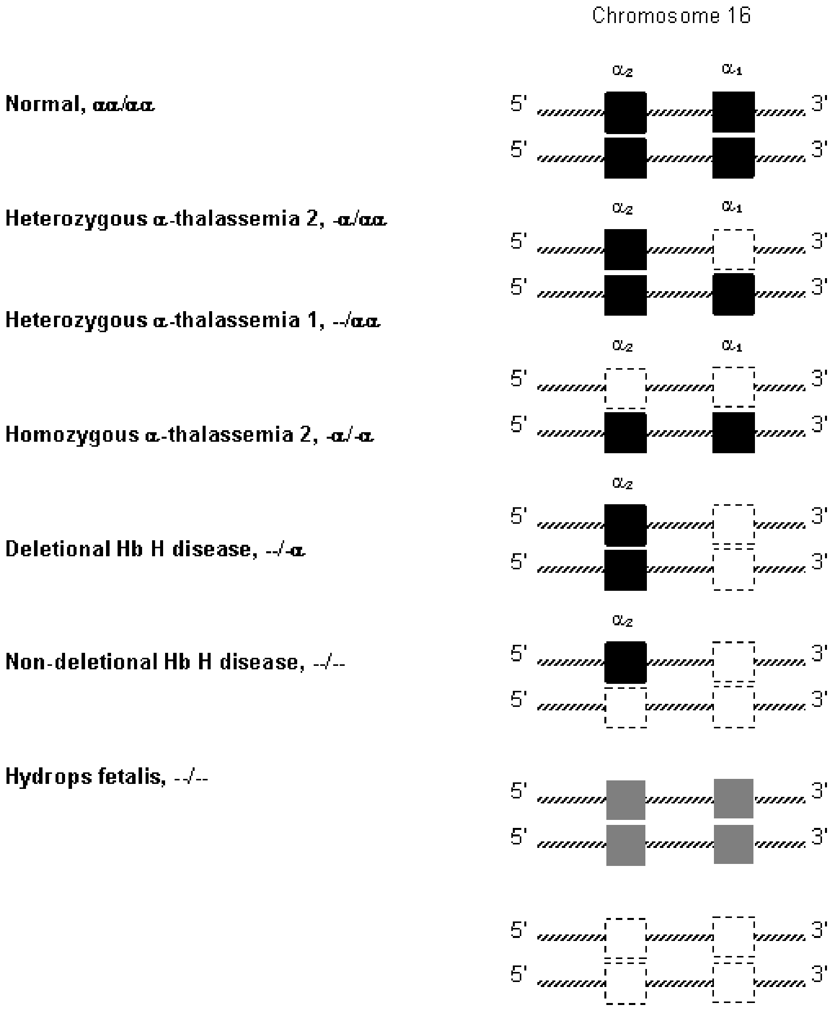

**FIGURE 2.** Diagramatic representation of α-thalassemia gene deletions. Normal genes are represented by solid black squares (■), gene deletions by open dashed squares (□), and variable gene expression is designated by solid grey squares (■).

**TABLE 1**
**α/β-Globin mRNA Ratio in Normal and α-Thalassemia Subjects**

| α-Globin Genotype | α/β Ratio |
|---|---|
| αα/αα | 1.3 |
| -α/αα | 0.8 |
| --/αα | 0.5 |
| -α/-α | 0.5 |
| --/-α | 0.2 |
| --/-- | 0 |

## GENETICS AND MOLECULAR CLASSIFICATION

The heterozygous state of thalassemia ($\alpha^+$) results from deletion of one α-globin gene (α-/αα). The most common deletions vary in length, 3.7 and 4.2 kb ($-\alpha^{3.7}$ and $-\alpha^{4.2}$). If both parents are carriers, it can be passed to an offspring, resulting in a two-gene deletion on the two chromosomes (α-/α-). The homozygous state of an α-thalassemia carrier occurs when both genes on the same chromosomes are deleted, named $\alpha^0$ (--/αα). This is referred to as *cis* deletion as opposed to *trans*, when the deletions are on opposite chromosomes. The $\alpha^0$-thalassemia is also described by the length of the deletion that removes both α genes; the length being different in the various deletions. The more common deletions are the SEA deletion and the Mediterranean (MED) deletion. The Philippine (FIL) and Thai (THAI) deletions are particularly long. There are more than 35 known deletional mutations; extensive descriptions of the various mutations have been published[11,14]. The high frequencies of the various α-thalassemias cause a major public health burden in the region of Southeast Asia[15].

The common two-gene mutations ($--^{SEA}$/αα) and ($--^{FIL}$/αα) are of particular importance due to the risk of causing Hb H disease when inherited along with a single-gene deletion or resulting in α-thalassemia major (Hb Bart's hydrops fetalis) when inherited from both parents. Based on the frequency of the $--^{SEA}$/αα carrier state, the incidence of Hb Bart's hydrops fetalis is expected to be between 0.5 and five per 1000 births, and Hb H disease between four and 20 per 1000 births[1]. The combination of one of these common two-gene deletions with a nondeletion form of α-thalassemia, in particular the Constant Spring allele, is also common. The Constant Spring results from a mutation in the "stop codon" (α142, Term-->Gln, TAA-->CAA in α2), which leads to the insertion of the amino-acid glutamine instead of termination of the chain synthesis, causing an elongated α-chain. This results in a greater degree of globin imbalance and a preferential binding of the mutated globin chain to the red cell membrane, causing its rigidity and instability. The cells are overhydrated, which manifests in a higher mean corpuscular volume (MCV) than seen in Hb H disease (66.1–68.0 vs. 57.7–64.8 fl).

Hb H disease (--/-α), caused by three α gene deletions, is the result of combined $\alpha^0$ and $\alpha^+$ so that only one functional α gene remains (Fig. 2). Frequently, the common $\alpha^0$ deletion ($--^{SEA}$) combined with an $\alpha^+$ mutation ($-\alpha^{3.7}$ and $-\alpha^{4.2}$) is the genotype of Hb H in Southeast Asia. The reduced α-globin chain synthesis results in the formation of tetramers by free β-globin chains (β4), Hb H. These tetramers have poor oxygen delivery capacity due to their high oxygen affinity. It is an unstable hemoglobin that, when oxidized, precipitates inside the red blood cells (RBC)[16,17]. The oxidative cellular and membrane damage results in shortened RBC survival[18]. In some nondeletional Hb H disease, the variant hemoglobin, such as Hb Quang Sze or Hb Constant Spring, is even less stable, causing additional membrane dysfunction and hemolysis[19]. Hemolysis is a major cause of anemia in α-thalassemia. In addition, the intracellular precipitants occurring in the developing erythrocyte can result in ineffective erythropoiesis, although to a much lesser extent than in β-thalassemia.

In the extreme deletion of all four genes (Hb Bart's hydrops fetalis syndrome), there is no α-chain synthesis and, therefore, no Hb F, A, or A2 synthesis. Most of the hemoglobin present is Hb Bart's, which has very poor capacity for oxygen delivery to the tissues. When some Hb Portland ($\zeta 2\gamma 2$) is produced, it is capable of delivering oxygen to the fetal tissues until the third trimester. However, in cases of homozygosity for the large --FIL or --THAI mutations that involve the ζ-genes, there is no Hb Portland synthesis, which results in an even worse syndrome, and earlier fetal death or miscarriage.

Although most α-globin mutations are of the deletional type, more than 40 nondeletional forms of α-thalassemia have been reported, causing deficient gene expression[20]. The carrier state (heterozygous) generally does not exhibit hemolytic anemia, having a minimal or mild clinical importance[21]. Others are unstable variants (for example, Hb Quong Sze) and cause a more severe hemolytic anemia[22]or have a more remarkable effect on oxygen affinity (Hb Chesapeake)[23].

The interaction of the different deletional and nondeletional mutations can produce a variety of phenotypes. Generally, the presence of a nondeletional mutation has a more severe effect on the α-globin gene expression, resulting in less compensatory expression of the remaining α genes and more unstable hemoglobin. Therefore, coinheritance of $\alpha^0$ and $\alpha^+$ deletions has a milder clinical phenotype than when an $\alpha^0$ deletion interacts with a nondeletional form[6,24,25]. Still, even within a particular genotype, there is a variation in the degree of anemia, a phenomenon not well understood.

## CLINICAL PRESENTATIONS AND MANAGEMENT

### Silent Carrier and α-Thalassemia Minor

One or two α gene deletions are clinically asymptomatic, although they are associated with various degrees of microcytosis and erythrocytosis. $\alpha^0$-Thalassemia, a two-gene deletion (--/αα), is characterized by a very mild anemia (within 1.0–1.5 g/dl of normal). Low MCV, mean corpuscular hemoglobin concentration (MCHC), and mean corpuscular hemoglobin (MCH), as well as increased RBC, are also more prominent in $\alpha^0$-thalassemia than in $\alpha^+$ genotype[24]. The microcytosis is occasionally assumed to be the result of nutritional iron deficiency and is treated as such. This can result in unnecessary or potentially harmful iron supplementation.

### Hb H Disease

Due to the instability of Hb H, affected patients have increased hemolysis and a mild to moderate anemia, as well as marked microcytosis and hypochromia. The levels of hemoglobin, MCV, and MCH are variable, but the overall range is 8.8–11.1 g/dl, 62–67 fl, and 19 pg, respectively[6]. In a steady state, most patients have no obvious clinical symptoms related to their disease and, if not diagnosed through neonatal screening, they may frequently present only after a complication has occurred. These complications primarily include anemia, gallstones, and jaundice[26]. The hemoglobin level can occasionally drop significantly, which is observed in children more often. Such severe anemia can occur during a febrile disease, an infectious episode, or due to exposure to oxidant drugs, causing increased hemolysis. Anemia can also be caused by a viral-induced transient aplasia, particularly with Parvovirus B19. Intermittent red cell transfusion may be needed and is reported in 41% of patients in a large series in China[26], but the requirement of regular transfusions is rare. However, correlation of the transfusion need to the mean hemoglobin level and to episodes of anemia was not examined. Growth retardation or delayed puberty is generally not a feature of Hb H disease, but has been reported in 13% of the cases in the same report.

Splenomegaly and, less commonly, hepatomegaly are frequently found. Due to the increased hemolysis, there is also a higher incidence of gallstones, reported in 15–34%[26,27]. A less common complication is the development of postsplenectomy thrombosis, particularly deep vein thrombosis, frequently involving the portal vein[28]. It is thought that the postsplenectomy thrombocytosis and intravascular hemolysis, a prominent feature of this form of thalassemia, contribute to the hypercoagulable state and thrombus formation. It is therefore recommended to use an anticoagulant in patients immediately following surgery and to proceed with long-term prophylaxis.

Iron overload is common in nontransfused patients with $\beta$-thalassemia mutations due to a higher rate of ineffective erythropoiesis than is generally seen in $\alpha$-thalassemia. Still, several studies have shown iron overload in Hb H disease, noted in over 70% of patients of adult age, caused by increased intestinal iron absorption stimulated by increased hemolysis and erythropoiesis[26,29,30]. In two studies, men had higher ferritin levels than females[31,32], while a third study found no relation to gender or to deletional vs. nondeletional genotype[26]. The severity correlated with age and seemed worse in splenectomized patients. Mean ferritin level over 900 µg/ml was observed in 17/23 adults in China who responded well to the oral chelator Deferiprone (L1), with reduction of liver iron and improved diastolic dysfunction on echocardiogram[33]. Other studies have shown a parallel increase in liver iron (by CT scan or MRI methods), suggesting that routine screening of nontransfusion-dependent Hb H patients can identify high-risk patients in whom early therapeutic intervention may prevent further complications and morbidity. The concern of underdiagnosed end-organ damage is demonstrated through findings of diastolic dysfunction on echocardiogram in 25 patients with $\alpha$-thalassemia, all with normal ejection fraction and no clinical cardiac symptoms[26].

Studies screening for iron overload–induced endocrinopathies are not reported in $\alpha$-thalassemia. Chim et al. reported a case of iron-induced diabetes mellitus in an adult with Hb H-Constant Spring[34]. There are no reports of compromised fertility in Hb H disease. However, during pregnancy, some women experience a fall of hemoglobin to the range of 7 g/dl or less, occasionally requiring transfusions[35]. A higher rate of premature labor (12%), pre-eclampsia, and congestive heart failure during the third trimester was reported[36]. It is important to follow pregnant women closely in conjunction with the perinatology specialist. Attention needs to be given to iron and folate status as an additional cause for anemia.

Ineffective erythropoiesis and the process of subsequent development of low bone mass is a feature that characterizes $\beta$-thalassemia mostly. A recent study also underscored this less-recognized complication among patients with Hb H and Hb H-Constant Spring. Spine Z scores of $-0.97 \pm 0.8$ and $-1.54 \pm 0.8$, respectively, and an overall fracture rate of 2.3–2.5%, were reported[37].

## Hb H–Constant Spring

Hb H-Constant Spring is a common nondeletional $\alpha$-thalassemia resulting from the combination of $\alpha^0$ and a Constant Spring (CS) structural mutation on one $\alpha$ gene of the other chromosome (--/-$\alpha^{cs}$). The homozygote state of Hb H-CS generally presents as milder anemia; however, reports of a more severe hemolysis and a potential predisposing cause for acute hemolysis were described as well as a case of severe fetal anemia with hydropic features[38,39]. It is difficult to detect the Hb H-CS on electrophoresis because of the very low Hb H-CS levels, and the reduced and unstable amounts of $\alpha$CS mRNA[40]. Some cases of Hb H-CS disease are therefore initially assumed to have deletional $\alpha$-thalassemia mutations (Hb H), and may escape the need for more proper education and monitoring of their disease[41,42]. DNA-based technology is therefore required. Clinically, patients with Hb H-CS have a more severe anemia, splenomegaly, cholelithiasis, and frequent episodes of fall in hemoglobin, due to a higher sensitivity to oxidant stimulus[41]. Moreover, the North American cross-sectional study for thalassemia showed that a third required regular transfusions[4]. The severity of this genotype has resulted in a mandatory screening for it by molecular technology in California[43].

Treatment for patients with Hb H disease and particularly for Hb H-CS needs to include education on potential complications and early monitoring for complications. In infants and young children, instructions for prompt attention to febrile disease, increased pallor and lethargy, and splenomegaly should be given, as aplastic or hemolytic episodes are not uncommon. Folic acid supplementation and avoidance of oxidative compounds and medications is an important part of early family education. As patients advance into adolescence and early adulthood, attention to the development of cholelithiasis, iron overload, and changes in bone density need to be monitored and treated[26]. Genetic counseling and education about the most severe forms of α-thalassemia should be provided. Thrombosis prevention is indicated in cases that undergo splenectomy and symptoms related to the presence of gallstones should be recognized. Attention should be given to patients (in particular those with Hb H-CS or other nondeletional thalassemia) who have more severe symptoms related to their anemia, as they might benefit from the initiation of regular transfusion therapy.

## Hb Bart's Hydrops Fetalis Syndrome

Hb Bart's hydrops fetalis syndrome, the most severe form of α-thalassemia, results from deletion of all four α-globin genes. If both parents carry the deletion of both α genes in *cis*, there is a 25% chance in each pregnancy for the fetus to inherit both mutations and lack all four genes. More rarely, it is due to coinheritance of $\alpha^0$- and $\alpha^+$-thalassemia or homozygosity for the Constant Spring mutation[39,44,45,46]. Four-gene deletion is the most common form of fetal hydrops in Southeast Asia[47,48] and has been increasingly recognized in other parts of the world over the past 2 decades[49].

Complications during pregnancy of a fetus carrying such mutations are well known and include pregnancy-induced hypertension and toxemia, antepartum hemorrhage, malpresentation, prematurity, and fetal distress[13]. The absence of the major fetal hemoglobin ($\alpha 2\gamma 2$), due to the total absence of α-globin synthesis, results in the fetus surviving primarily on Hb Bart's ($\gamma 4$) and Hb Portland ($\zeta 2\gamma 2$). Hb Bart's, which constitutes about 80% of the hemoglobin in the affected fetus, is an unstable hemoglobin with poor oxygen delivery to the tissues, consequently resulting in severe anemia, tissue hypoxia, heart failure, extramedullary hematopoiesis, edema, and placental complications. Hb Portland (10–20% of the affected fetal hemoglobin) is more effective in oxygen delivery, allowing fetuses to survive until the third trimester.

Surviving fetuses are usually born prematurely and have a large variability of the spectrum of clinical presentations. There is moderate to severe anemia (usually 3–8 g/dl) and many, but not all, are grossly hydropic. Most newborns have high-output cardiac failure with cardiomegaly, pleural and pericardial effusions, and general edema. The edema results from cardiac failure and hypoalbuminemia, which is secondary to poor liver function, affected by extramedullary hematopoiesis. A high percentage of neonatal anomalies have been described including hydrocephalus, microcephalus, abnormal genitalia, limb reduction defects, as well as hypoplastic lungs, kidneys, and adrenal glands. Typically, the placenta is significantly enlarged and friable[24].

Carrier detection based on a low MCV and subsequent DNA analysis for couples at risk is essential for proper genetic counseling. Consequent prenatal diagnosis can be done effectively with current DNA-based technology[50]. This can reduce the risk of maternal morbidity and the incidence of hydrops fetalis if couples choose to terminate the pregnancy. Ultrasound examination can detect changes in the placenta and abnormal fetal cardiothoracic ratio consistent with hydrops early in pregnancy[51]. This noninvasive procedure was shown to make the correct diagnosis in a high percentage of cases[52] and to be the diagnostic method of choice for many couples[53]. Examining fetal erythrocytes in the maternal circulation that carry only the embryonic ζ-globin chain may also prove to be an accurate noninvasive method for diagnosis of a pregnancy carrying a Hb Bart's hydrops fetus[54]. Recently, preimplantation genetic diagnosis (PGD) was successfully performed with PCR methods for exclusion of homozygous α-thalassemia and implantation of unaffected embryos[55].

Over the past 2 decades, an increasing number, at least 15 cases, of surviving children with homozygous α-thalassemia was reported. These were usually transfused immediately after birth or received intrauterine transfusions. The subsequent treatment consisted of regular transfusions and iron chelation[13,49], and in some cases, a bone marrow transplant was performed[56]. As a result of these advances in technology, it is likely that more parents are pursuing active treatment instead of terminating the pregnancy, raising medical and ethical questions on the long-term outcome of these patients. Indeed, more recent reports have looked at the outcome of such babies: Lee et al.[57] reviewed 11 cases who survived after receiving intensive care without prior intrauterine therapy; five of them had abnormal neurological outcomes including developmental delay and spastic quadriplegia. An approximate 50% neurological or developmental problems was also described by Lucke et al.[58]. In contrast, several case reports discuss affected fetuses who were treated with intrauterine transfusions, resulting in the birth of nonhydropic babies who had only minor or no abnormalities, and "normal growth and development"[56,59,60,61].

However, studies testing the long-term neurodevelopmental outcome in these children are needed. Such studies were performed in children who were treated with intrauterine transfusions for immune hemolytic anemia. A review of 18 cases of hydropic fetuses due to severe immune hemolytic anemia treated with early intrauterine transfusions showed a high survival rate (89%) and favorable neuropsychological outcome at 10 years of age. Twenty-two percent (4/18) died or had a major neurological sequela[62]. This study suggests a favorable long-term outcome in patients with immune as well as nonimmune hydrops fetalis, and emphasizes the benefit of early intervention with intrauterine transfusions. Hb Bart's α-thalassemia fetuses are likely affected earlier and more severely than fetuses with immune hemolytic anemia; therefore, such comparison should be taken cautiously. Still, these studies provide important and encouraging data on the efficacy of intrauterine treatment and could assist families dealing with this dilemma.

## SCREENING FOR CARRIER STATUS: PRESENT AND FUTURE

The α-thalassemia trait should be diagnosed in the case of microcytosis (MCV < 80 fl) with a normal hemoglobin A2 level (<3.5%), in the absence of iron deficiency. Accurate diagnosis of the α-globin genotype is required for genetic counseling and prenatal diagnosis. In cases of microcytosis due to both α and β mutations (with an elevated A2 level), the diagnosis of an α-thalassemia carrier can be missed if the blood count findings are only attributed to the β mutation and DNA analysis is not performed. Prenatal diagnosis is indicated when parental screening suggests a risk of hydrops fetalis, but not when Hb H disease is suspected. Early diagnosis is crucial for avoiding later maternal complications. DNA diagnosis from amniocentesis is usually performed, but noninvasive methods are available as discussed in the section on hydrops fetalis.

Antenatal screening programs are implemented in many countries and reliably detect the β-thalassemia trait; however, they cannot exclude the α-thalassemia trait since the α genotype is not examined. Detection of couples at risk for a child with homozygous α-thalassemia is a major aim in many screening programs and requires accurate PCR-based genetic diagnosis and, when relevant, prenatal diagnosis. Although available, the high cost has hindered wide-range implementation in countries in Southeast Asia. In a study implementing molecular screening, it was found that it was not superior to screening for microcytosis to identify individuals heterozygous for the $\alpha^0$-thalassemia genotype in the ethnic groups at risk[63]; however, in populations where both β and α mutations are prevalent, avoiding molecular techniques can result in missing the diagnosis of fetuses affected by serious α-thalassemia syndromes. Overall, genetic screening utilizing multiplex PCR-based methods is frequently beyond the means of countries in Southeast Asia. Financial support of major international agencies, and the cooperation of governments and communities of Eastern countries with Western countries, are crucial for implementing diagnostic programs, as previously proposed[1,64].

Screening of newborns for Hb H disease was implemented in California in 1999[65] and expanded to include confirmatory testing by DNA analysis for detection of Hb H-CS[43]. Initial screening has expanded to include a program aimed to resolve ambiguous results from State Newborn Hemoglobinopathy Screening[66]. The determination of an early diagnosis allows proper care for these infants and raises the awareness of screening for the prevention of homozygous α-thalassemia.
